# Supplementary material for: China’s GDP forecasting using Long Short Term Memory Recurrent Neural Network and Hidden Markov Model
Source: PLoS One. 2022 Jun 17;17(6):e0269529. doi: 10.1371/journal.pone.0269529 (PMC9205526; doi:10.1371/journal.pone.0269529)
Supplement: S1 Appendix — (PDF) [file pone.0269529.s001.pdf]

S1 Appendix. In the appendix, we give a brief explanation of Viterbi algorithm involved in this paper referring to [22], and take the decoding process of HMM in The benchmark model: HMM as an example of using Viterbi algorithm, where we find the most likely hidden state sequence that explains the observations.

We represent the parameters of HMM involved in the decoding process with $\lambda = (A, B)$. Given λ and a sequence of observations $V = \{v_1, \cdots, v_T\}$ with N kinds of states, we need to find the most probable hidden state sequence $S = \{s_1, \cdots, s_T\}$. We apply the Viterbi algorithm to find the states that best explains the observations. We do not need to keep a complete record of how we arrived at a certain state but record one previous cell using a trellis, which is equivalent to a Dynamic Programming table with states j as rows and time steps t as columns. For each cell, we calculate the Vierbi probability of reaching each state once for each time step, which is the probability of the optimal state sequence ending in state q_j at time t and represented as $\delta_j(t)$:

$$\delta_j(t) = \max_{s_0, \cdots, s_{t-1}} P(s_0 \cdots, s_{t-1}, v_1, \cdots, v_t, S_t = q_j|\lambda) \tag{12}$$

where we have $\delta_j(1) = a_{0j} b_j(x)$ at time step 1 that stores the probability of moving to state s_j from the start state and having emitted v_1. We then calculate recursively the Viterbi probability by taking the most probable of the extensions of the paths that lead to current $\delta_j(t)$:

$$\delta_j(t) = \max_{0 \leq i \leq N} \delta_i(t-1) \cdot a_{ij} \cdot b_j(x_t) \tag{13}$$

which comprises the previous path probability $\delta_i(t-1)$, the transition probability a_{ij} and the emission probability $b_j(x_t)$ and is calculated by maximising over the best ways of transferring to s_j for each s_i.

We define $\psi_j(t)$ to store the $t-1$ state index on the highest probability path, which is used to find the previous cell in the best path in the backtracing phase.

$$\psi_j(t) = arg \max_{0 \leq i \leq N} \delta_j(t-1) a_{ij} b_j(x) \tag{14}$$

Then for the final time step T, we have

$$P(V|X, \mu) = \delta_f(T+1) = \max_{1 \leq i \leq N} \delta_i(T) a_{ij} \tag{15}$$

which represents the probability of the entire state sequence up to final point $T+1$ having been produced given the observation and the HMM's parameters, where there is no $b_j(x)$ for the final state s_t does not emit. Accordingly, we have

$$\psi_f(T+1) = arg \max_{1 \leq i \leq N} \delta_i(T) \cdot a_{ij}^{'} \tag{16}$$

which records the last state at time T of the best path. We will then go back to the cell concerned and look up its ψ to find the second-but-last state and backtrace the path in the same way. We apply the Viterbi algorithm to the decoding process of HMM involved in each model, while the observation states vary with different classifiers respectively. We select the CPI fluctuation states obtained from CPI series with one phase lag to be the observable state of HMM and GMM-HMM, while we select the CPI fluctuation states obtained from predicted CPI series using LSTM to be the observable state of LSTM-HMM. The decoding process above can be applied to all HMM within each model involved in this paper.
