# Supplementary material for: China’s GDP forecasting using Long Short Term Memory Recurrent Neural Network and Hidden Markov Model
Source: PLoS One. 2022 Jun 17;17(6):e0269529. doi: 10.1371/journal.pone.0269529 (PMC9205526; doi:10.1371/journal.pone.0269529)
Supplement: Attachment — Submitted filename: Sub-Response to reviewers-2021-08-02(2).pdf [file pone.0269529.s002.pdf]

Responses to Reviewers

China's GDP Forecasting using Long Short Term Memory Memory Recurrent Neural Network and Hidden Markov Model

We really appreciate the helpful comments from the reviewers. We provide detailed responses to each of the comments as follows:

Reviewer ♯1 COMMENT 1:

The authors should consider reducing the number of abbreviations in the abstract, and properly define some of them along with the abstract (and article), such as GDP and CPI.

Reply: Thank you very much for the comment. We have supplemented the full name of GDP and CPI in abstract. The revised part is given as follows.

Revised part on page 1:

This paper presents a Long Short Term Memory Recurrent Neural Network and Hidden Markov Model (LSTM-HMM) to predict China's Gross Domestic Product (GDP) fluctuation state within a rolling time window. We compare the predictive power of LSTM-HMM with other dynamic forecast systems within different time windows, which involves the Hidden Markov Model (HMM), Gaussian Mixture Model-Hidden Markov Model (GMM-HMM) and LSTM-HMM with an input of monthly Consumer Price Index (CPI). ......

Reviewer ♯1 COMMENT 2:

The innovation point of the article is not clear enough. In the abstract and in the discussion it is not very clear what are the findings, and more important why might that be relevant?

Reply: Thank you very much for the comment. We have supplemented the innovation point in the introduction part. There are three innovative points for this paper, which range from the application methods of LSTM in LSTM-HMM, the selection of inflation rate indicator CPI as the input of GDP fluctuation states forecast and the findings of suitable time window for rolling prediction and the usefulness of LSTM-HMM in forecasting GDP fluctuation states. The revised

part about innovation is on page 3. As for the findings of this paper, we give a comprehensive review of results in experiments across time windows and models, which is shown in Table 14 and Fig. 12. We find from these results that among all the time windows, models within 8-year time window have better overall performance in accuracy and consistence and LSTM-HMM with an input of monthly CPI generally has good precision, and within 8-year time window it has the best accuracy and consistency. The two most important reasons are that (1) 8-year window with 32 observations in each round of training is long enough for us to observe all types of GDP fluctuation states and also short enough to avoid the bias caused by too many observations of certain type; (2)LSTM take more effects of historical CPI into the training process and tend to predict a suitable real time CPI fluctuation state, which replaces the lagged observable states adopted by other models, and helps in the prediction of GDP fluctuation using HMM. The revised part is on page 25. All the revised parts are given as follows.

Revised part on page 3:

Motivated by the usefulness of HMM and LSTM in economic forecast and the sparsity of literatures on the application of LSTM-HMM in GDP forecast, this paper establishes a LSTM-HMM to predict GDP fluctuation states. We further compare the predictive power of LSTM-HMM with HMM and GMM-HMM using monthly CPI or quarterly CPI within different time windows. There are three innovation points. First, in LSTM-HMM, we innovatively utilise LSTM to predict real-time CPI fluctuation states while feeding this prediction into the forecast of real-time GDP fluctuation states. Second, we select the inflation indicator CPI as the model input and utilise available monthly and quarterly CPI respectively in the LSTM-HMM. Third, we find from the empirical analysis of China's GDP fluctuation states that among all the time windows of rolling prediction, models within 8-year time window have better overall performance in accuracy and consistence and LSTM-HMM with an input of monthly CPI generally has good precision, which has the best accuracy and consistency with an input of monthly CPI within 8-year time window.

Revised part on page 25 and page 26:

......There are several reasons for these model results: firstly, 8-year window with 32 observations in each round of training is long enough for us to observe all types of GDP fluctuation states and also short enough to avoid the bias caused by too many observations of certain type as a proper data source for LSTM-HMM; secondly, LSTM-HMM take more effects of historical CPI into the training process with the application of LSTM and tend to predict a suitable

real time CPI fluctuation state, which replaces the lagged observable states adopted by other models, and helps in the prediction of GDP fluctuation using HMM;

Reviewer ♯1 COMMENT 3:
What is the motivation to introduce a LSTM?

Reply: Thank you very much for the comment. This is a very helpful suggestion. LSTM is a key component of LSTM-HMM, which is characterized with the capability of processing tasks involving long time lags. There are studies on the economic forecast that utilise LSTM (Zhang and Huang , 2021; Zahara et al , 2020), while there are also researches on the application of HMM in economic forecast (Gregoir and Lenglart, 2000; Bellone and Gautier, 2004). However, there isn't any research on GDP forecast that utilises LSTM-HMM, which involves the LSTM's prediction of observable states for HMM. Therefore, this motivates our research that tries to combine the potentials of LSTM and HMM. We have detailed the motivation of introducing LSTM to HMM on page 2 and page 3. The revised parts are as follows:

Revised part on page 2:
Gross Domestic Product (GDP) is a key indicator of economic growth, which measures the total value of goods and services produced within a country in a year, not including its income from investments in other countries. There are considerable literatures on economic prediction in financial market that utilises Hidden Markov Model (HMM) (Gregoir and Lenglart, 2000; Bellone and Gautier, 2004) and Long Short-Term Memory (LSTM) recurrent neural network (Zhang and Huang , 2021; Zahara et al , 2020). However, there isn't any study on the application of LSTM-HMM in GDP forecast that combines the potentials of these two models, which serves as the motivation of our research on the China's GDP forecast using LSTM-HMM based on the dynamic relationship between inflation and economic growth.

Revised part on page 3:
Long Short-Term Memory (LSTM) is a type of recurrent neural network that works better on tasks involving long time lags by bridging huge time lags between relevant input events (Ronald J and Jing, 1990), which has emerged as an effective and scalable model for time-series prediction. LSTM is applied to CPI prediction in Indonesia with multivariate input (Zahara et al , 2020). LSTM is also utilised to the optimal hedging in the presence of market frictions (Zhang and Huang , 2021), which shows usefulness in the empirical analysis of real option markets.

Motivated by the usefulness of HMM and LSTM in economic forecast and the sparsity of literatures on the application of LSTM-HMM in GDP forecast, this paper establishes a LSTM-HMM to predict GDP fluctuation states ......

Reviewer ♯1 COMMENT 4:

In section 2.1, I found the notation confusing. The authors write that "S is a discrete set (...), where t stands for time.", but I do not see t before that. After this phrase, the authors start to use s_t, is this the same capital S defined before?

Thank you very much for the comment. We modified the definition of discrete set S, which is a time series denoted with time t. The revised part is as follows:

Revised part on page 4:
...... $S = \{s_1, s_2, ..., s_t, ...\}$ is a discrete set of GDP fluctuation states, where t stands for time. ......

Reviewer ♯1 COMMENT 5:

In Section 3.1, when describing Fig. 5 the authors mention "the curve" and "the straight line". I suppose this is a typo.

Reply: Thank you very much for the comment. To tell the difference of GDP and CPI growth rate, we replace Fig.5 with the new figure, where GDP growth rate is represented with solid line, CPI growth rate is represented with dotted line. The revised part is as follows:

Revised part on page 10: (See Fig.1)

We firstly describe the trend of annual data of CPI and GDP to have a general understanding about their similarity of growth rate. The trend of annual GDP growth rate and CPI growth rate is shown in Fig.1, where the dotted line represents the annual growth rate of CPI and the solid line represents the annual growth rate of GDP.

Reviewer ♯1 COMMENT 6:

In section 3.2, the Granger does not exactly measure causality, therefore it is better to use the term Granger predictive causality, and clarify this in the text.

Reply: Thank you very much for the comment. The revised part about it is as follows:

[1] Data source: China's National Bureau of Statistics http://www.stats.gov.cn/

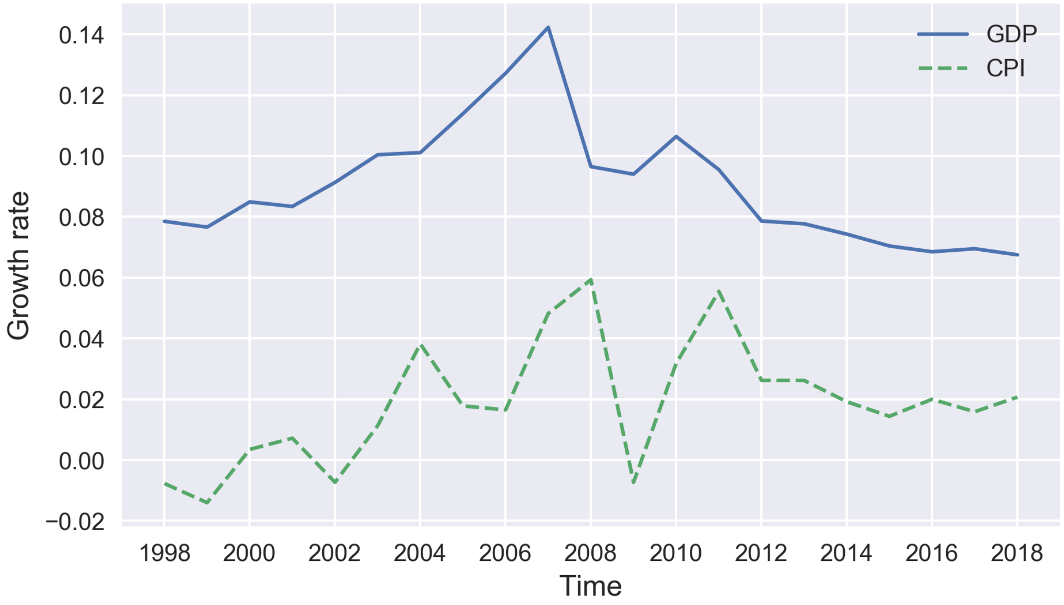

Figure 1: The trend of GDP-CPI growth rate[1]

Revised part on page 11:

We conduct a test of Granger predictive causality with x_t and y_t to obtain the interaction relationship between CPI and GDP in the same period. Considering correlation between two variables that indicates comovement, Granger causality relates to the idea of incremental predictive power of one time series for forecasting another time series, which is a statistically testable criterion based on the ideas of precedence and predictive power (Croux and Reusens, 2013; Yao et al, 2000). ......

Reviewer ♯1 COMMENT 7:

The results of the article are based on the comparison of numbers without any clear interpretation. Many of the numerical results have four to five decimal digits (e.g. γ). Is this precision really significant? For instance, the authors draw conclusions and compare models with an accuracy of 0.6406 and 0.6563. Is this difference really significant?

Reply: Thank you very much for the comment. We have revised the number of decimal digits as 2 for uniform and significant description, since Bellone and Gautier (2004) set the number of decimal digits as 2 while conducting economic downturn analysis and HMM prediction. The

revised parts are as follows:

Revised part on page 11: (See Table.1)

Table 1: The linear regression analysis of quarterly year-on-year GDP growth rate and quarterly year-on-year CPI growth rate.

Variables	Coefficients	S.E.	T-value	P-value
β_0	72.49	10.85	6.68	***
β_1	0.27	0.10	2.74	**
Residual S.E.: 1.90	D.F.:79	P-value: ***		

As we can see from Table 1, the coefficient β_0 is estimated to be 72.49 and β_1 is estimated to be 0.27, which reach the significance level of 5% and show a positive correlation between y_t and x_t, where for every unit change of CPI growth rate, the average change of CPI growth rate is 0.27 units. The degree of freedom represented as D.F. in Table 1 is 79 with 80 samples and one independent variable. Residual S.E. is 1.90. The model has a good goodness of fit with high overall significance since its P-value is only less than 0.01. We then test the stability of the residuals e_t of the linear model to avoid spurious regression using unit root test, where the P-value with lag order of 4 is below the 5 % significance level. The result of unit root test shows the stability of the time series e_t involved in our analysis, and we then conduct a cointegration test using method proposed by (Phillips and Ouliaris, 1990), which shows that under the significance level of 10 percent, we can reject the hypothesis of no cointegration relationship since the statistical value is 29.83, higher than the critical value of 27.43 at right tail.

Revised part on page 12: (See Table 2)

Table 2: The result of correlation analysis

Indexes	$x_t v.s. h_t$	$y_t v.s. h_t$
T-value	15.88	0.80
D.F.	79	79
Correlation	0.88	0.09
P-value	***	0.43

[1]Standard errors are in parentheses. Significance: $^{***}p < 0.01$; $^{**}p < 0.05$; $^{*}p < 0.1$.

As we can see from Table 2, the correlation of x_t and y_t is 0.88 with a P-value less than 1 %.

Revised part on page 13:

The 90% confidence intervals under different scales are also plotted(the dotted line) respectively, so we can read off the asymptotic 90% confidence set [101.33,102.67] from the graph from where test statistics cross the dotted line. where $\hat{\gamma}$ is significant when taking the value of 101.37.

Revised part on page 14:(See Table 3)

Table 3: Least squares estimates of 2-regime threshold model

Parameters	Coefficients	S.E.	C.I.
α_1	77.95	45.20	[-10.65,166.55]
θ_1	0.32	0.45	[-0.57,1.20]
α_2	45.30	12.93	[19.96,70.65]
θ_2	0.62	0.12	[0.37,0.86]

As we can see from Table 3, the model allows the slope parameters to differ depending on the value of h_t. The slope coefficient θ_1 is 0.32 given $h_t \leq 101.37$, while the slope coefficient θ_2 is 0.62 given $h_t \geq 101.37$. ......

Reviewer ♯2 COMMENT 1:

Throughout the text the author describes several properties without specifying what CPI means. Therefore, the author must need to define and make clear what CPI means.

Reply: Thank you very much for the comment. We have supplemented the meaning of CPI in abstract while describing CPI as a key indicator of inflation rate in introduction part. The revised parts are as follows:

Revised part on page 1:

This paper presents a Long Short Term Memory Recurrent Neural Network and Hidden Markov Model (LSTM-HMM) to predict China's Gross Domestic Product (GDP) fluctuation state within a rolling time window. We compare the predictive power of LSTM-HMM with

other dynamic forecast models within different time windows, which involves the Hidden Markov Model (HMM), Gaussian Mixture Model-Hidden Markov Model (GMM-HMM) and LSTM-HMM with an input of monthly Consumer Price Index (CPI). ......

Revised part on page 2:

...... Among these researches, Consumer Price Index (CPI) has been utilised as the indicator of inflation rate (Sarel, 1996; Hwang and Wu, 2011), while Gross Domestic Product (GDP) is also a key indicator economic growth (Sarel, 1996; Gerlach-Kristen, 2009). ......

Reviewer ♯2 COMMENT 2:

In the last paragraph of the Conclusion Section the author states that "In practical application, we should try our best to reduce the impact of selection bias.". The author should clarify what he means by "practical application" and what kinds of selection bias is he talking about.

Reply: Thank you very much for the comment. We supplement the explanation about selection bias and also replace practical application with more detailed explanation. The revised parts are as follows:

Revised part on page 26:

..... In the empirical analysis of GDP fluctuation states forecast, models encapsulated with HMM tend to ignore the effect of the sparse states and the giant difference of numbers of states will result in selection bias. In this paper, we reduce the impact of selection bias by experiments within pre-selected lengths of time window ranging from 4-year, 6-year, 8-year to 10-year and find that within 8-year window, the model performance is better. ......

Reviewer ♯2 COMMENT 3:

The author must be revise all figures and tables of the manuscript, with clear description in the respective captions, in order to improve the quality and readability of the manuscript. For instance, there is a typo in Table 13.

Reply: Thank you very much for the comment. We have detailed the captions of figures and tables. The revised parts are as follows:

Revised part on page 4:(See Fig.2)

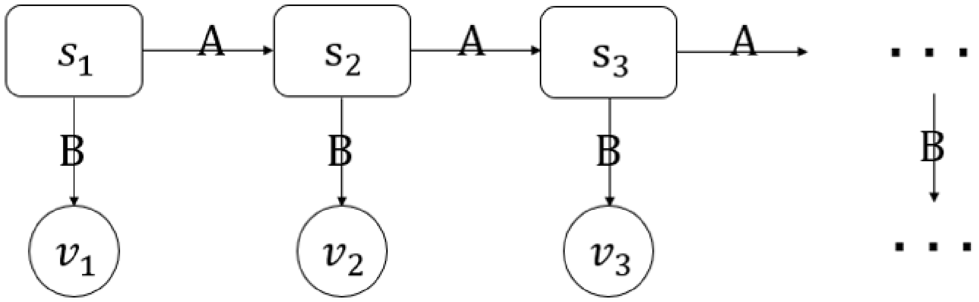

Figure 2: Structure of the Hidden Markov Model (HMM) that we build as the benchmark

Revised part on page 7:(See Fig.3)
Revised part on page 8: (See Fig.4 and Fig.5)
Revised part on page 11:(See Table 4)

Table 4: The result of Granger causality test of quarterly year-on-year GDP growth rate and quarterly year-on-year CPI growth rate.

H_0	Lag period	Sample size	F-value	P-value
y_t is not the cause of x_t	5	80	2.578	0.04*
x_t is not the cause of y_t	2	80	9.79	***

Revised part on page 14:(See Table 5)

Table 5: The threshold estimates from two-stage least squares estimation for the threshold model

Threshold estimate $\hat{\gamma}$	101.37
C.I. - Uncorrected	[101.33,102.67]
C.I. - Het Corrected 1	[101.33,102.67]
C.I. - Het Corrected 2	[101.33,102.67]

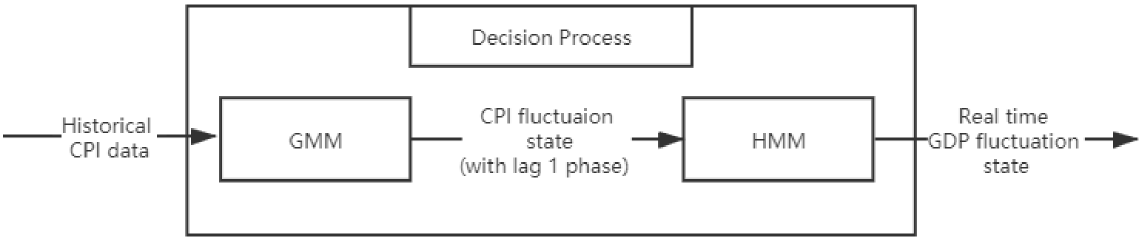

Figure 3: The decision process of the Gaussian Mixture Model-Hidden Markov Model (GMM-HMM) that introduces Gaussian Mixture Model (GMM) to transform CPI series into a categorical sequence.

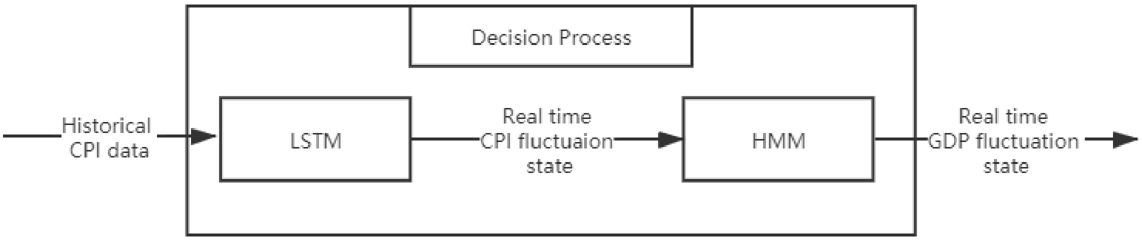

Figure 4: The whole decision process of Long Short Term Memory Recurrent Neural Network and Hidden Markov Model (LSTM-HMM) with quarterly univariate-input that introduces LSTM to forecast CPI fluctuation states

Revised part on page 15:(See Table 6 and Table 7)

Table 6: GDP fluctuation states that differ in three types

Sequence value	GDP fluctuation state
0	Low-speed growth stage
1	Appropriate-speed growth stage
2	High-speed growth stage

Table 7: A general criteria for CPI fluctuation states with two different types

Sequence value	Confirmation standard	CPI fluctuation state
0	$x \leq 101.37$	Moderate inflation
1	$x > 101.37$	Intensified inflation

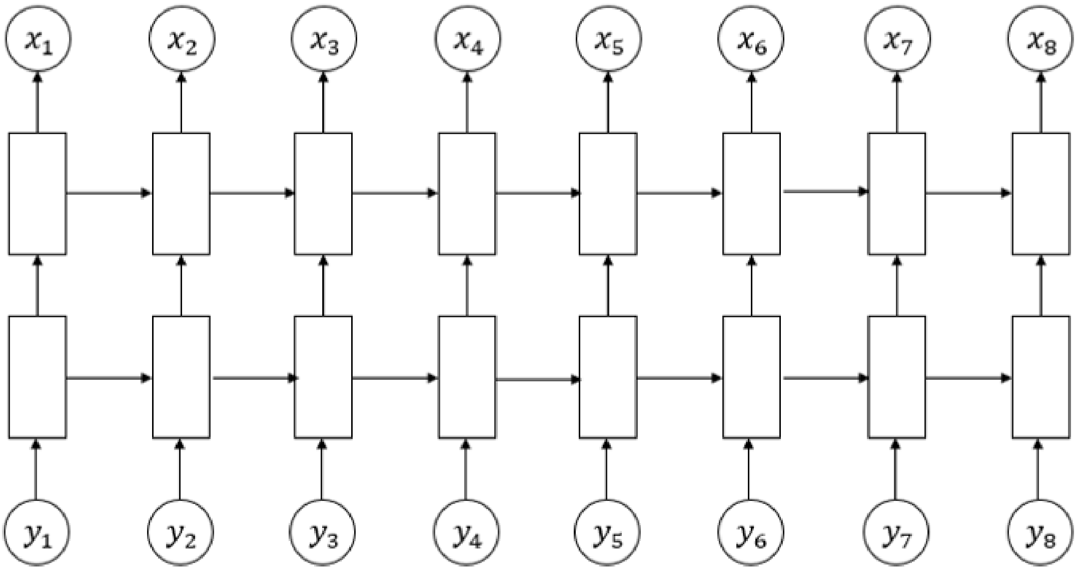

Figure 5: The structure of the LSTM we build to predict CPI fluctuation states using historical CPI series.

Revised part on page 16:

Table 8: Criteria of CPI fluctuation states for HMM with monthly input that vary in four types

Sequence value	Confirmation standard	CPI fluctuation state	Feature
0	$x_{t1} \leq 101.37$	$x_{t2} \leq 101.37$	Moderate-Moderate inflation
1	$x_{t1} \leq 101.37$	$x_{t2} > 101.37$	Moderate-Intensified inflation
2	$x_{t1} > 101.37$	$x_{t2} \leq 101.37$	Intensified-Moderate inflation
3	$x_{t1} > 101.37$	$x_{t2} > 101.37$	Intensified-Intensified inflation

Revised part on page 18:(See Fig.6)

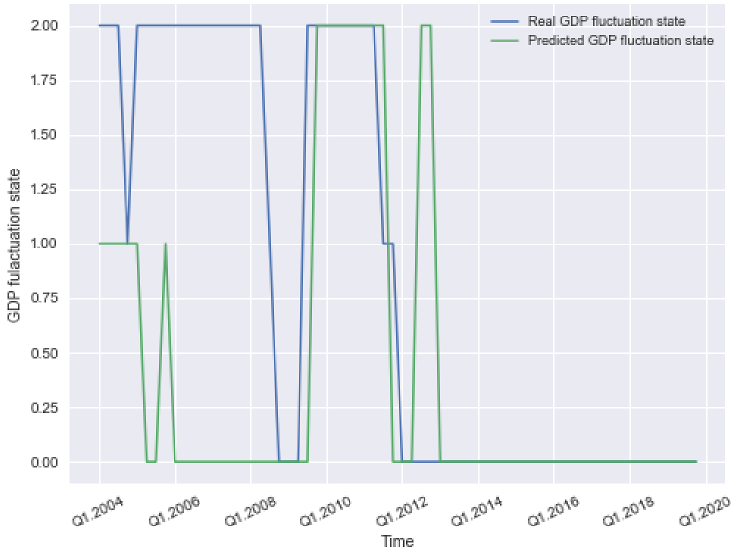

Figure 6: The predictions of GDP fluctuation states using HMM(q) and 4-year time window

Revised part on page 19: (See Table 9)

Table 9: The number of observations and predictions of GDP fluctuation states within 4-year, 6-year, 8-year and 10-year time window

Time window	Forecasting period	Observations(Q)	Observations(M)	Predictions
4-year (2000-2003)	16-year (2004-2019)	16	32	64
6-year (2000-2005)	14-year (2006-2019)	24	48	56
8-year (2000-2007)	12-year (2008-2019)	32	64	48
10-year (2000-2009)	10-year (2010-2019)	40	80	40

Revised part on page 20:(See Fig.7 and Table 10)

Table 10: Accuracy, Kappa and AUC for HMM, GMM-HMM and LSTM-HMM with monthly or quarterly input and 4-year time window

	HMM(q)	GMM-HMM(q)	LSTM-HMM(q)	HMM(m)	GMM-HMM(m)	LSTM-HMM(m)
Accuracy	0.64	0.56	0.66	0.61	0.64	0.64
Kappa	0.31	0.14	0.34	0.25	0.32	0.30
AUC	0.73	0.67	0.74	0.71	0.73	0.73

Revised part on page 21:(See Fig.8 and Table 11)

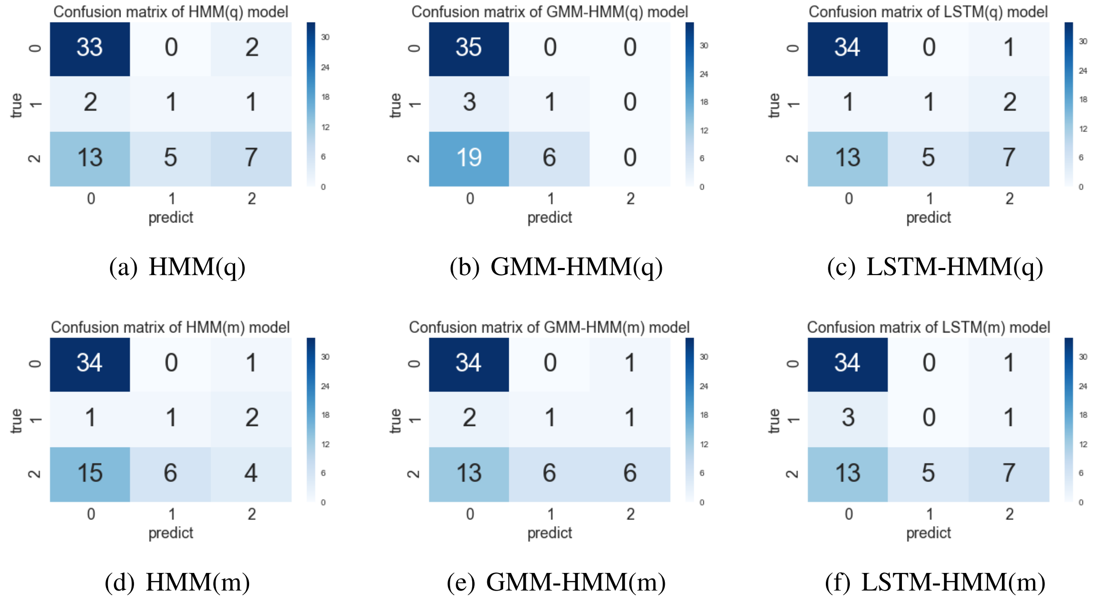

(a) HMM(q) (b) GMM-HMM(q) (c) LSTM-HMM(q)

(d) HMM(m) (e) GMM-HMM(m) (f) LSTM-HMM(m)

Figure 7: Confusion matrixes for prediction results from HMM, GMM-HMM and LSTM-HMM with monthly or quarterly input and 4-year time window

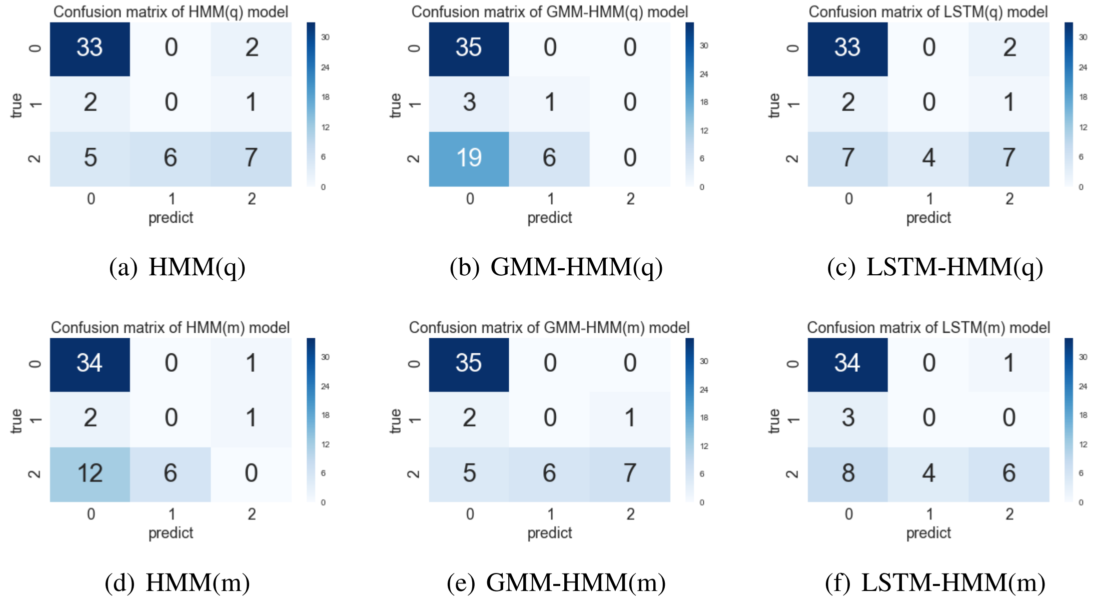

(a) HMM(q) (b) GMM-HMM(q) (c) LSTM-HMM(q)

(d) HMM(m) (e) GMM-HMM(m) (f) LSTM-HMM(m)

Figure 8: Confusion matrixes for prediction results from HMM, GMM-HMM and LSTM-HMM with monthly or quarterly input and 6-year time window

Table 11: Accuracy, Kappa and AUC for HMM, GMM-HMM and LSTM-HMM with monthly or quarterly input and 6-year time window

	HMM(q)	GMM-HMM(q)	LSTM-HMM(q)	HMM(m)	GMM-HMM(m)	LSTM-HMM(m)
Accuracy	0.71	0.63	0.71	0.61	0.75	0.71
Kappa	0.42	0.14	0.39	0.12	0.48	0.37
AUC	0.79	0.72	0.79	0.71	0.81	0.79

Revised part on page 22: (See 9 and Table 12)

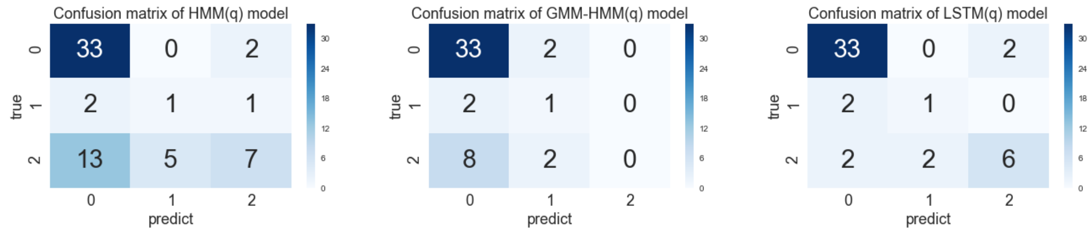

(a) HMM(q) (b) GMM-HMM(q) (c) LSTM-HMM(q)

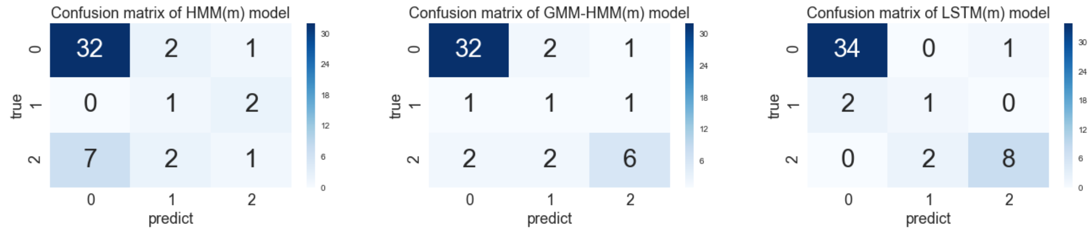

(d) HMM(m) (e) GMM-HMM(m) (f) LSTM-HMM(m)

Figure 9: Confusion matrixes for prediction results from HMM, GMM-HMM and LSTM-HMM with monthly or quarterly input and 8-year time window

Table 12: Accuracy, Kappa and AUC for HMM, GMM-HMM and LSTM-HMM with monthly or quarterly input and 8-year time window

	HMM(q)	GMM-HMM(q)	LSTM-HMM(q)	HMM(m)	GMM-HMM(m)	LSTM-HMM(m)
Accuracy	0.83	0.71	0.83	0.71	0.81	0.90
Kappa	0.62	0.14	0.58	0.24	0.56	0.75
AUC	0.88	0.78	0.88	0.78	0.86	0.92

Revised part on page 23:(See Fig.10)

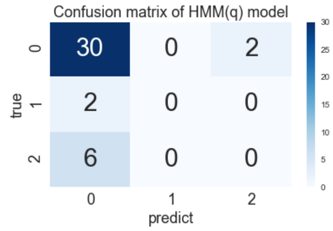

(a) HMM(q)

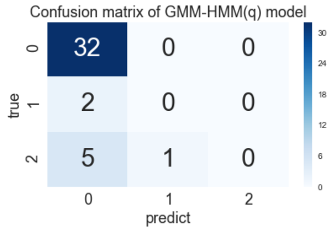

(b) GMM-HMM(q)

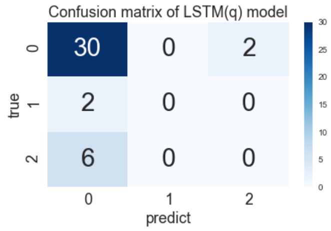

(c) LSTM-HMM(q)

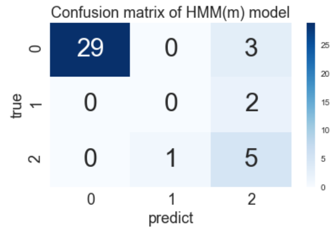

(d) HMM(m)

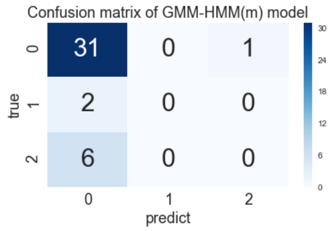

(e) GMM-HMM(m)

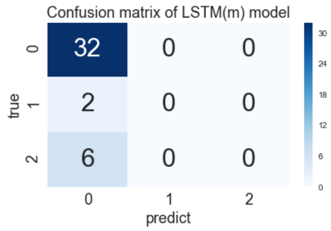

(f) LSTM-HMM(m)

Figure 10: Confusion matrixes for prediction results from HMM, GMM-HMM and LSTM-HMM with monthly or quarterly input and 10-year time window

Revised part on page 24:(See Table 13 and Fig.11)

Table 13: Accuracy, Kappa and AUC for HMM, GMM-HMM and LSTM-HMM with monthly or quarterly input and 10-year time window

	HMM(q)	GMM-HMM(q)	LSTM-HMM(q)	HMM(m)	GMM-HMM(m)	LSTM-HMM(m)
Accuracy	0.75	0.80	0.75	0.85	0.78	0.80
Kappa	-0.08	0.09	-0.08	0.61	-0.04	0.00
AUC	0.81	,0.85	0.81	0.89	0.83	0.85

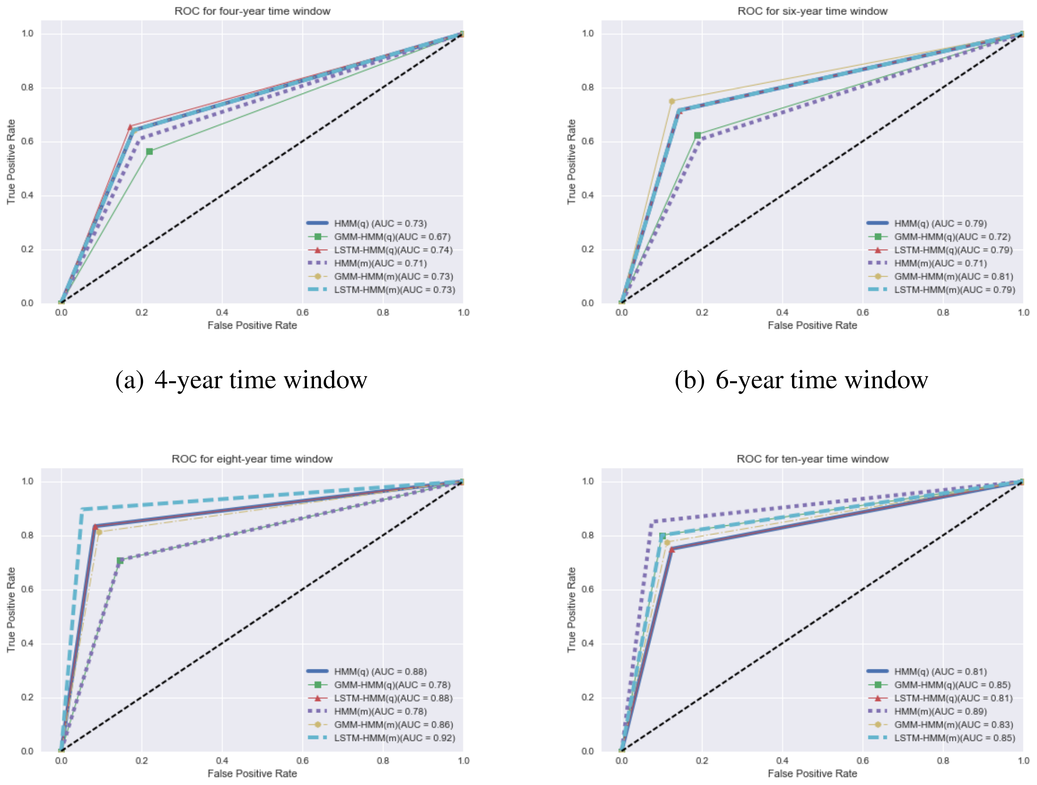

(a) 4-year time window

(b) 6-year time window

(c) 8-year time window

(d) 10-year time window

Figure 11: ROC for HMM, GMM-HMM and LSTM-HMM with monthly or quarterly input and 4-year, 6-year, 8-year or 10-year time windows

Revised part on page 25:(See Table 14)

Table 14: Comparing HMM, GMM-HMM and LSTM-HMM with monthly or quarterly input and 4-year, 6-year, 8-year or 10-year time windows based on Accuracy, Kappa and AUC

T.W.	HMM(q)	GMM-HMM(q)	LSTM-HMM(q)	HMM(m)	GMM-HMM(m)	LSTM-HMM(m)
4 year	(0.64, 0.31, 0.73)	(0.56,0.14,0.67)	(0.66,0.34,0.74)	(0.61,0.25,0.70)	(0.64,0.32,0.73)	(0.64,0.30,0.64)
6 year	(0.71,0.42, 0.79)	(0.63, 0,14,0.72)	(0.71,0.39,0.79)	(0.61,0.12,0.71)	(0.75,0.48,0.81)	(0.71, 0.37,0.79)
8 year	(0.83, 0.62,0.88)	(0.71,0.14,0.78)	(0.83,0.58,0.88)	(0.71,0.24,0.78)	(0.81,0.56,0.86)	(0.90,0.75,0.92)
10 year	(0.75,-0.08,0.81)	(0.80,0.09,0.85)	(0.75,-0.08,0.81)	(0.85,0.61,0.89)	(0.73,-0.0.04,0.83)	(0.80,0.00,0.85)

Reviewer ♯2 COMMENT 4:

Considering the results of the ROC curves, there is not a monotonic behavior in performance of the algorithms with the time window. Especially for 8-year time window, there

is a clear spread in the ROC curves. I think that the author must provide some indication as to why this is so.

Reply: Thank you very much for the comment. We have supplemented description of performance of ROC curves within each time window and tried to provide some indication of the reason. The revised part is as follows:

Revised part on page 24 and page 25:

For 4-year time window in Fig.11(a), the ROC curves do not vary much. Among the models, LSTM-HMM has better performance with both quarterly and monthly input. LSTM-HMM and HMM performs better with quarterly input, but GMM-HMM performs better with monthly input. For 6-year time window in Fig.11(b), there is a clear spread between ROC curves of HMM(q), LSTM-HMM(q), GMM-HMM(m) and LSTM-HMM(m) and ROC curves of GMM-HMM(q) and HMM(m). This spread may result from model input, as GMM-HMM with monthly input and 6-year time window performs better than GMM-HMM with quarterly input and 6-year time window, while for HMM with 6-year time window, the model with quarterly input performs better. For 8-year time window in Fig.11(c), there is also a clear spread between ROC curves, which is similar with the spread in 6-year time window, resulting from different model inputs. In addition, LSTM-HMM(m) performs best with highest AUC value. For 10-year time window in Fig.11(d), ROC curves share similar trends, where LSTM-HMM and HMM performs better with quarterly input, but GMM-HMM performs better with monthly input. We can see from Fig.11 that, within all the time windows, LSTM-HMM(m), GMM-HMM(m), LSTM-HMM(q) generally have good performances in prediction. The ROC curves of different models share similar trends within each time window.

References

Williams Ronald J and Peng Jing. An efficient gradient-based algorithm for on-line training of recurrent network trajectories. *Neural computation*, 2(4):490–501, 1990.

Junhuan Zhang and Wenjun Huang. Option hedging using LSTM-RNN: an empirical analysis. *Quantitative Finance*, 2021:1–20, 2021.

S Zahara, Suguanto and M B Ilmiddaviq Consumer price index prediction using Long Short

Term Memory (LSTM) based cloud computing. *Journal of Physics: Conference Series*, 1456 (1):012–022, 2020.

Christophe Croux and Peter Reusens. Do stock prices contain predictive power for the future economic activity? A Granger causality analysis in the frequency domain. *Journal of Macroeconomics*, 35:93–103, 2013.

Feng Yao and Yuzo Hosoya. Inference on one-way effect and evidence in Japanese macroeconomic data. *Journal of Econometrics*, 98(2):225–255, 2000.

Stephane Gregoir and Fabrice Lenglart. Measuring the probability of a business cycle turning point by using a multivariate qualitative hidden markov model. *Journal of Forecasting*, 19 (2):81–102, 2000.

Benot Bellone and Erwan Gautier. Predicting economic downturns through a financial qualitative hidden markov model. *Journal of Forecast*, 2004.

Peter CB Phillips and Sam Ouliaris. Asymptotic Properties of Residual Based Tests for Cointegration. *Econometrica*, 69(58):165–193, 1990.

Michael Sarel. Nonlinear effects of inflation on economic growth. *IMF Economic Review*, 43 (1):199–215, 1996.

Jente Hwang and Mingjia Wu. Inflation and economic growth in china: An empirical analysis. *China and World Economy*, 19(5):67–84, 2011.

Petra Gerlach-Kristen. Business cycle and inflation synchronisation in mainland china and hong kong. *International Review of Economics and Finance*, 18(3):404 – 418, 2009.
