# Supplementary material for: China’s GDP forecasting using Long Short Term Memory Recurrent Neural Network and Hidden Markov Model
Source: PLoS One. 2022 Jun 17;17(6):e0269529. doi: 10.1371/journal.pone.0269529 (PMC9205526; doi:10.1371/journal.pone.0269529)
Supplement: Attachment — Submitted filename: Response to reviewers-20220227-no-original-Zhang.pdf [file pone.0269529.s003.pdf]

Responses to Reviewers

China's GDP Forecasting using Long Short Term Memory Memory Recurrent Neural Network and Hidden Markov Model

We really appreciate the helpful comments from the reviewers. We provide detailed responses to each of the comments as follows:

Reviewer ♯1 COMMENT 1:

In section 3.2, the authors write: "...which shows that under the significance level of 10 percent, we cannot reject the hypothesis of no cointegration relationship since the statistical value is 29.83, higher than the critical value of 27.43 at right tail.". How do the authors think that cointegration may, or may not, affect the results found for their model?

Reply: Thank you very much for the comment. The cointegration test is employed to test the presence of cointegration relationship between multiple time series. The cointegration test here indicates the causality relationship between GDP growth rate and CPI growth rate. And this leads to the Granger predictive causality test between GDP growth rate and CPI growth rate. The revised part is given as follows.

Revised part on page 8:

$\cdots\cdots$ and we then conduct a cointegration test using method proposed by (Phillips and Ouliaris, 1990) for the presence of cointegration in the time series of CPI growth rate and GDP growth rate. The result shows that under the significance level of 10 percent, we can reject the hypothesis of no cointegration relationship since the statistical value is 29.83, higher than the critical value of 27.43 at right tail, which indicates the causality between y_t and x_t.

Reviewer ♯1 COMMENT 2:

In section 3.4, the authors define how the time series of GDP fluctuation will be discretized. This is one of the most important parts of the manuscript as it can affect the quality of the time series. Figures 8, 9 and 10 clearly show the confusion matrices generated from an unbalanced test set with many observations of 0's. Are training sets suffering from the

same problem? what are the impacts of choosing different dividing points on the quality of the training set?

Reviewer ♯1 COMMENT 3:

Still in section 3.4, the authors support the choice of division points based on a 2008 paper that uses data from 1990 to 2006 to define the turning points of the GDP growth rate curve. In this sense, in some time windows shown in Table 9, the models are using information from the future in their predictions. Have the authors tested adapting the choice of dividing points to time windows to avoid this kind of inconsistency?

Reply: Thank you very much for the Comment 2 and 3 in Section 3.4. The states of GDP fluctuation is defined based on the discretization of GDP growth rate. They are divided according to the turning points of the GDP growth rate series referring to the previous study conducted by (Liu, 2008), including the low-speed growth stage, the appropriate-speed growth stage and the high-speed growth stage. The three stages each represents different characteristics of GDP growth rate and indicates the differences of the GDP fluctuation range. Therefore, the turning points of GDP growth rate is taken as the dividing points of GDP fluctuation states.

We improve the GDP fluctuation states based the estimation of the turning points of GDP growth rate from 2000 to 2019, which covers the range of data set employed for the experiment. The threshold auto-regression model is used to estimate the number and the values of the turning points, where the result is tested using the eF test proposed by Caner and Hansen (2001). Based on the improved GDP fluctuation states, both the training set and the testing set have balanced observations with an improved model performance in the prediction of GDP fluctuation state 1 and 2. The revised parts, including the identification of the GDP fluctuation states and the improved model performance, are as follows:

Revised part on page 11-12:

$\cdots\cdots$ we use a threshold auto-regression model to estimate the turning points of the GDP growth rate curve between 2000 and 2019. We represent the GDP growth rate at time t with y_t and set the corresponding threshold variable as $z_t = y_{t-d}$ with a delay order $d(d \leq p)$. The TAR model is defined as

$$\begin{aligned} y_t = (\beta_{10} + \beta_{11} y_{t-1} + \cdots + \beta_{1p} y_{t-p} + \sigma_1 \varepsilon_t) I_{(z_t \leq c_1)} \\ +(\beta_{20} + \beta_{21} y_{t-1} + \cdots + \beta_{2p} y_{t-p} + \sigma_2 \varepsilon_t) I_{(c_1 < z_t \leq c_2)} \\ +(\beta_{30} + \beta_{31} y_{t-1} + \cdots + \beta_{3p} y_{t-p} + \sigma_1 \varepsilon_t) I_{(z_t > c_3)} \end{aligned}$$

where the c_1 and c_2 each represents the turning points of GDP growth rate. We set p as 8 and estimate the parameters including the delay order d, the turning points c_1 and c_2, as well as the coefficients of the variables. The results are shown in Table1

Table 1: The results of the threshold auto-regression model of the GDP growth rate

	Regime 1 ($z_t \leq 107.50$)		Regime 2 ($107.50 < z_t \leq 108.90$)		Regime 3 ($z_t > 108.90$)	
Variable	Estimate	S.E.	Estimate	S.E.	Estimate	S.E.
Constant	-63.93 ***	20.50	-116.72 ***	34.59	5.43	13.01
y_{t-1}	0.73 ***	0.17	-0.20	0.21	1.21***	0.14
y_{t-2}	0.61 ***	0.12	0.48 ***	0.13	-0.43	0.24
y_{t-3}	-0.19	0.16	0.35**	0.12	0.08	0.33
y_{t-4}	-1.29 ***	0.17	-0.02	0.20	-0.08	0.30
y_{t-5}	1.29 ***	0.26	0.39	0.27	0.05	0.24
y_{t-6}	0.58	0.34	0.12	0.21	-0.05	0.25
y_{t-7}	-0.93 ***	0.17	-0.33***	0.11	-0.27	0.22
y_{t-8}	0.80 ***	0.19	1.29***	0.38	0.42**	0.15
	Delay Order d	8	SSE	40.94	F value	36.70

As we can see from Table 1, the delay order p is estimated to be 8. The values of the threshold are estimated as 107.5 and 108.9. The sum of squared errors of the estimated TAR is 40.94. Referring to Caner and Hansen (2001), the test for the null hypothesis of a one-regime autoregression (AR) against the alternative hypothesis of a three-regime TAR are conducted. The F value is calculated as 36.69, higher than the 99% significance level, indicating the rejection of the null hypothesis of AR.

We divide the state of economic fluctuation into low-speed growth stage, appropriate-speed growth stage and high-speed growth stage with the turning points of the GDP growth rate curve as the dividing points, which is 7.50% and 8.90% respectively.

Revised part on page 13:

$\cdots\cdots$ The emission probability matrix is shown as follows:

$$\begin{pmatrix} 0.14 & 0.86 \\ 0.35 & 0.65 \\ 0.46 & 0.54 \end{pmatrix}$$

where given GDP fluctuation state taking different values, the CPI fluctuation states are more likely to take value 1, which means that the fluctuation of CPI series is not moderate in most

cases. The emission probabilities of CPI taking value 1 given different GDP fluctuation states vary, while the emission probability of CPI fluctuation states taking value 1 given GDP fluctuation state of 0 is the highest among other emission probabilities within the matrix.

......

The emission probability matrix is shown as follows:

$$\begin{pmatrix} 0.04 & 0.11 & 0.14 & 0.71 \\ 0.35 & 0.00 & 0.00 & 0.65 \\ 0.37 & 0.03 & 0.06 & 0.54 \end{pmatrix}$$

where given GDP fluctuation state taking different values, the CPI series are more likely to be in the stage of "Intensified-Intensified inflation". Given GDP fluctuation states taking the value of 1, CPI series are more likely to be in the stage of "Moderate-Moderate inflation" and "Intensified-Intensified inflation" with no samples taking the value of 1 or 2.

Revised part on page 14:

We decode the HMM structure using observations from 2000 to 2019,······ Fig.1 below shows the predicted values and the real values.

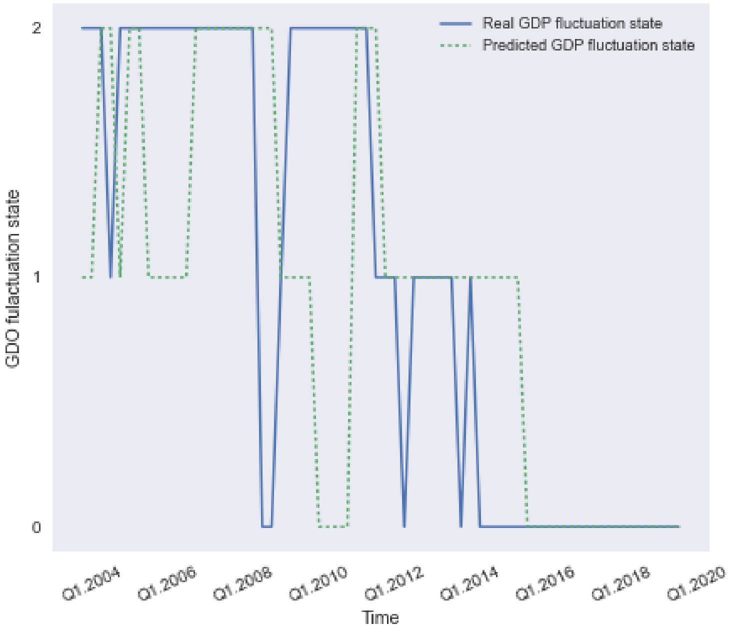

Figure 1: The predictions of GDP fluctuation states using HMM(q) within 4-year time window

As wee can see from Fig.1, there are 64 predictions in total, ranging from 2004.Q1 to 2019.Q4. There are 41 predictions coinciding with the real values, where there are 17 accurate predictions of GDP fluctuation states with the value of 0, 9 accurate predictions of GDP fluctuation states of value 1 and 12 accurate predictions of GDP fluctuation states of value2, while there are 26 wrong predictions evenly distributed. The predictions after 2012 have a better performance than the predictions before that time and the two curves in Fig.1 fit with each other. To better evaluate the prediction capability of the forecasting models, we then train the other models using various methods based on data from different time windows.

Revised part on pages 15-16:

We run the six models including HMM(q) with quarterly input, GMM-HMM(q) with quarterly input, LSTM-HMM(q) with quarterly input, HMM(m) with monthly input, GMM-HMM(m) with monthly input and LSTM-HMM(m) with monthly input within 4-year time windows and their confusion matrixes calculated based on the predictions and real values are shown in Fig.2.

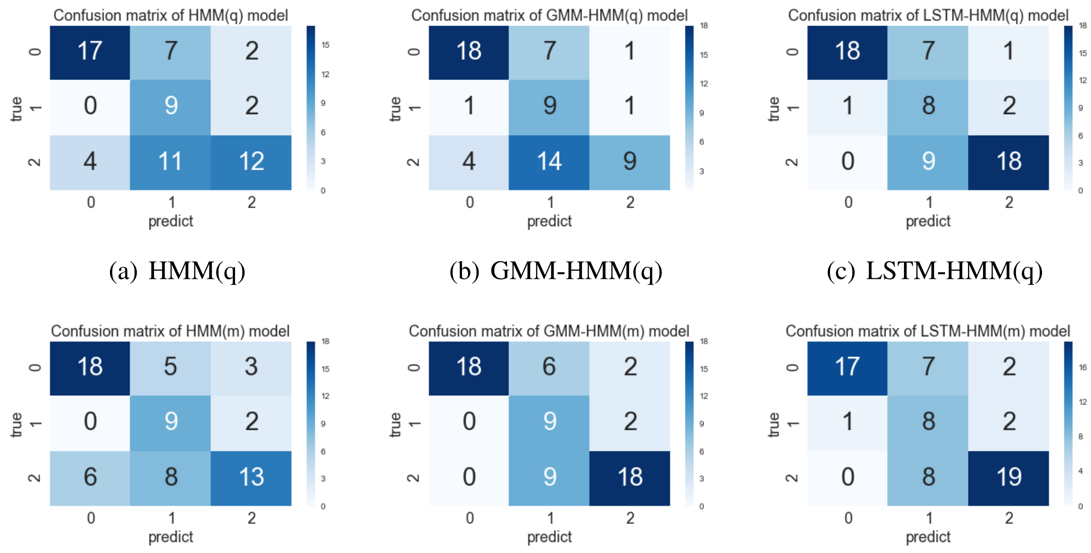

(a) HMM(q) (b) GMM-HMM(q) (c) LSTM-HMM(q)

(d) HMM(m) (e) GMM-HMM(m) (f) LSTM-HMM(m)

Figure 2: Confusion matrixes for prediction results from HMM, GMM-HMM and LSTM-HMM with monthly or quarterly input and 4-year time window

As we can see from the Fig.2, there are 64 predictions in total, where there are many predictions of GDP fluctuation states with value 0 and 2. All these models have precise predictions of

GDP fluctuation states with value 0, while GMM-HMM with monthly input and LSTM-HMM with either monthly or quarterly input also have good performance in the prediction of GDP fluctuation state 1. From the perspective of overall efficiency, we can see that GMM-HMM with monthly input has 45 accurate predictions. This is more than that of other predictions and is slightly better than the performance of LSTM-HMM. Based on the confusion matrixes shown in Fig.2, we calculated the accuracy vector necessary for the evaluation, including accuracy, kappa and AUC, where these details are shown in Table 2. The models with monthly input are denoted with m, while the models with quarterly input are denoted with q.

Table 2: Accuracy, Kappa and AUC for HMM, GMM-HMM and LSTM-HMM with monthly or quarterly input and 4-year time window

	HMM(q)	GMM-HMM(q)	LSTM-HMM(q)	HMM(m)	GMM-HMM(m)	LSTM-HMM(m)
Accuracy	0.59	0.56	0.69	0.63	0.70	0.69
Kappa	0.41	0.38	0.54	0.44	0.56	0.54
AUC	0.70	0.67	0.77	0.72	0.78	0.77

As is shown in Table 2, the GMM-HMM with monthly input has the highest prediction accuracy, which is 0.70, with the highest kappa of 0.56 and the highest AUC of 0.78, showing a moderate consistency and a good performance in prediction. HMM(m), LSTM-HMM(q) and LSTM-HMM(m) also perform better than random judgement, with a prediction accuracy over 0.60, while HMM(q) and GMM-HMM(q) do not have a satisfactory performance in prediction, with a lower prediction accuracy, kappa and AUC. When the model input transforms from quarterly to monthly, the model performance of the HMM and GMM-HMM can be improved in terms of accuracy, kappa and AUC, while the LSTM-HMM, with either monthly or quarterly input, keeps the same model performance.

To sum up, within the 4-year time window which involves 16 observations for each round of training, the GMM-HMM(m) with quarterly input has the best performance, while the HMM(q) and GMM-HMM(q) with quarterly input may not be applicable to the prediction in this condition.

Revised part on pages 16 and 17:

We run the six models including HMM(q), GMM-HMM(q), LSTM-HMM(q), HMM(m), GMM-HMM(m) and LSTM-HMM(m) within 6-year time windows and their confusion matrixes are shown in Fig.3.

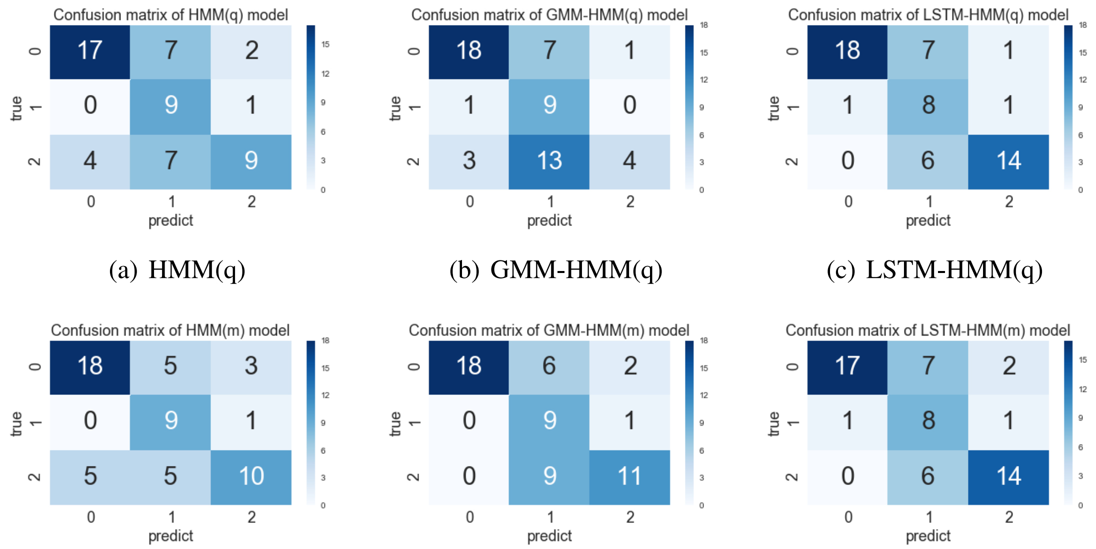

(a) HMM(q) (b) GMM-HMM(q) (c) LSTM-HMM(q)

(d) HMM(m) (e) GMM-HMM(m) (f) LSTM-HMM(m)

Figure 3: Confusion matrixes for prediction results from HMM, GMM-HMM and LSTM-HMM with monthly or quarterly input and 6-year time window

As we can see from the Fig.3, there are 56 predictions in total, and the characteristics of these confusion matrixes are similar with that of models within 4-year time window. All these models predict many GDP fluctuation states with value 0. The GMM-HMM(m), LSTM-HMM(q), LSTM-HMM(m) have relatively better performance in the prediction of GDP fluctuation state 2. From the perspective of overall efficiency, we can see that LSTM-HMM(q) and LSTM-HMM(m) have more accurate predictions. Based on the confusion matrixes shown in Fig.3, we calculated the accuracy vector necessary for the evaluation and the details are shown in Table 3.

Table 3: Accuracy, Kappa and AUC for HMM, GMM-HMM and LSTM-HMM with monthly or quarterly input and 6-year time window

	HMM(q)	GMM-HMM(q)	LSTM-HMM(q)	HMM(m)	GMM-HMM(m)	LSTM-HMM(m)
Accuracy	0.63	0.55	0.71	0.66	0.68	0.70
Kappa	0.45	0.36	0.58	0.49	0.53	0.55
AUC	0.72	0.67	0.79	0.75	0.76	0.77

As is shown in Table 3, the LSTM-HMM(m) has the highest accuracy rate, which is 0.71, with the highest kappa of 0.58 and the highest AUC of ~~0.81~~0.79, showing a moderate consistency with the real values and a good performance in prediction. The LSTM-HMM(m)

also has good performance in prediction, with an accuracy rate of 0.70. While the GMM-HMM(q),HMM(q) and HMM(m) have a lower prediction accuracy, kappa and AUC. When the model input transforms from quarterly to monthly, the model performance of the HMM and GMM-HMM can be improved in terms of accuracy, kappa and AUC, while the LSTM-HMM, with either monthly or quarterly input, have similar model performance.

To sum up, within the 6-year time window which involves 24 observations for each round of training, the LSTM-HMM(q) with monthly input has the best performance.

Revised part on page 17:

We run the six models including HMM(q), GMM-HMM(q), LSTM-HMM(q), HMM(m), GMM-HMM(m) and LSTM-HMM(m) within 8-year time windows and their confusion matrixes are shown in Fig.4.

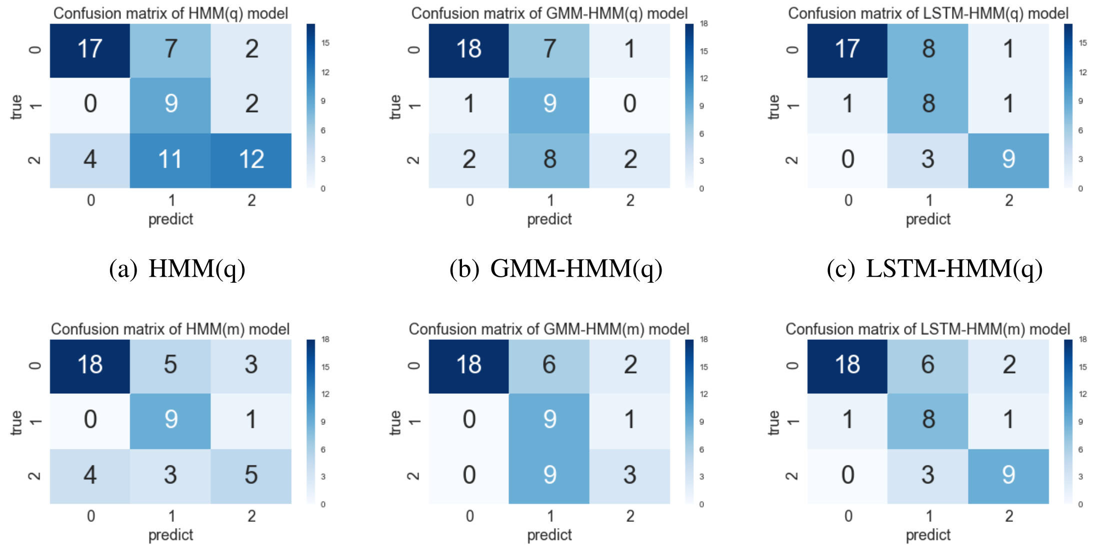

(a) HMM(q) (b) GMM-HMM(q) (c) LSTM-HMM(q)

(d) HMM(m) (e) GMM-HMM(m) (f) LSTM-HMM(m)

Figure 4: Confusion matrixes for prediction results from HMM, GMM-HMM and LSTM-HMM with monthly or quarterly input and 8-year time window

As we can see from the Fig.4, there are 48 predictions in total, and the characteristics of these confusion matrixes are similar with that of models within 4-year time window and 6-year time window. All these models have accurate predictions of GDP fluctuation states of value 0. The LSTM-HMM with monthly input has the best performance since it has 35 precise predictions

among the 48 predictions. Based on the confusion matrixes shown in Fig.4, we calculated the accuracy vector necessary for the evaluation in Table 4.

Table 4: Accuracy, Kappa and AUC for HMM, GMM-HMM and LSTM-HMM with monthly or quarterly input and 8-year time window

	HMM(q)	GMM-HMM(q)	LSTM-HMM(q)	HMM(m)	GMM-HMM(m)	LSTM-HMM(m)
Accuracy	0.65	0.60	0.71	0.67	0.63	0.73
Kappa	0.45	0.38	0.56	0.47	0.43	0.58
AUC	0.73	0.70	0.78	0.75	0.72	0.80

As is shown in Table4, LSTM-HMM(m) with monthly input has the highest prediction accuracy, which is 0.73, with the highest kappa of 0.58 and the highest AUC of 0.80. LSTM-HMM(m) has a moderate consistency with the real values and a better performance in prediction than that of HMM(q). HMM(m), GMM-HMM(q) and GMM-HMM(m). LST<-HMM(q) also have good performances in prediction with an accuracy rate of over 0.71. When the model input transforms from quarterly to monthly, the model performance of the HMM, GMM-HMM and LSTM-HMM can be improved in terms of accuracy, kappa and AUC.

To sum up, within the 8-year time window which involves 32 observations for each round of training, the LSTM-HMM(m) with monthly univariate-input has the best performance, while HMM(q) and GMM-HMM(m) also perform well in prediction.

Revised part on page 17 and 18:

We run the six models including HMM(q), GMM-HMM(q), LSTM-HMM(q), HMM(m), GMM-HMM(m) and LSTM-HMM(m) within 10-year time windows and their confusion matrixes are shown in Fig.5.

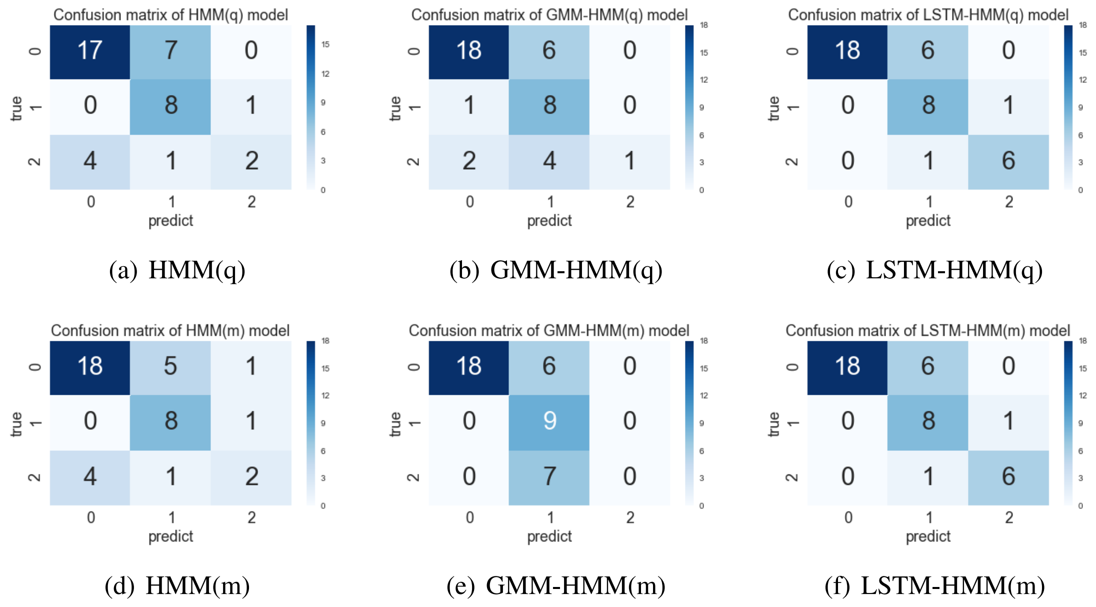

(a) HMM(q) (b) GMM-HMM(q) (c) LSTM-HMM(q)

(d) HMM(m) (e) GMM-HMM(m) (f) LSTM-HMM(m)

Figure 5: Confusion matrixes for prediction results from HMM, GMM-HMM and LSTM-HMM with monthly or quarterly input and 10-year time window

As we can see from the Fig.5, there are 40 predictions in total, and the characteristics of these confusion matrixes are different with that of models in the above subsections. All these models have relatively precise predictions, where ther are many predictions of GDP fluctuation state 0 but a smaller number of predictions of GDP fluctuation state 2. ······ We then try to evaluate them according to the accuracy parameters shown in Table 5.

Table 5: Accuracy, Kappa and AUC for HMM, GMM-HMM and LSTM-HMM with monthly or quarterly input and 10-year time window

	HMM(q)	GMM-HMM(q)	LSTM-HMM(q)	HMM(m)	GMM-HMM(m)	LSTM-HMM(m)
Accuracy	0.68	0.68	0.80	0.70	0.68	0.80
Kappa	0.44	0.44	0.67	0.48	0.46	0.67
AUC	0.76	0.76	0.85	0.78	0.76	0.85

As is shown in Table 5, the LSTM-HMM with monthly univariate-input has the highest accuracy rate, which is 0.80, and the highest AUC of 0.85, while the kappa is 0.67, which shows that the predictions of this model have substantial consistency. When the model input transforms from quarterly to monthly, the model performance of the HMM, GMM-HMM and LSTM-HMM keep the same or have small improvement in terms of accuracy, kappa and AUC.

To sum up, within the 10-year time window which involves 40 observations for each round of training and 40 predictions, the LSTM-HMM with monthly univariate input has the best performance in prediction, while the other models also perform well in the prediction of GDP fluctuation states.

Revised part on pages 18 and 19:

We compare their ROC curves within different time windows, which are shown below in Fig.6.

For 4-year time window in Fig.6(a), there is a spread between ROC curves, where GMM-HMM(m), LSTM-HMM(q) and LSTM-HMM(m) performs better than the other models. For 6-year time window in Fig.6(b), the ROC curves are similar with the ROC curves of 4-year time window, while there is a larger spread between the ROC of GMM-HMM(q) and that of others. This may result from the CPI classifier and the quarterly input, as GMM-HMM with monthly input and HMM with quarterly input have better performance in terms of ROC. For 8-year time window in Fig.6(c), there is a clear spread between the ROC curves of LSTM-HMM and the ROC curves of the other models, which shows an improved model performance with the proposed LSTM-HMM. For 10-year time window in Fig.6(d), ROC curves share similar trends with a even larger spread between the ROC curves of LSTM-HMM and that of others. We can see from Fig.6 that, within all the time windows, LSTM-HMM(m) and LSTM-HMM(q) generally have better performances in prediction than the other models. The ROC curves of different models share similar trends within each time window.

Table 6: Comparing HMM, GMM-HMM and LSTM-HMM with monthly or quarterly input and 4-year, 6-year, 8-year or10-year time windows based on Accuracy, Kappa and AUC

T.W.	HMM(q)	GMM-HMM(q)	LSTM-HMM(q)	HMM(m)	GMM-HMM(m)	LSTM-HMM(m)
4 year	(0.59,0.41,0.70)	(0.56,0.38,0.67)	(0.69,0.54,0.77)	(0.63,0.44,0.72)	(0.70,0.56,0.78)	(0.69,0.54,0.77)
6 year	(0.63,0.45,0.72)	(0.55,0.36,0.67)	(0.71,0.58,0.79)	(0.66,0.49,0.75)	(0.68,0.53,0.76)	(0.70,0.55,0.77)
8 year	(0.65,0.455,0.73)	(0.60,0.38,0.70)	(0.71,0.56,0.78)	(0.67,0.47,0.75)	(0.63,0.43,0.72)	(0.73,0.58,0.80)
10 year	(0.68,0.44,0.76)	(0.68,0.44,0.76)	(0.80,0.67,0.85)	(0.70,0.48,0.78)	(0.68,0.46,0.76)	(0.80,0.67,0.85)

We also compare their accuracy vector: (accuracy, kappa, AUC) shown in Table 6, where we find that from the perspective of time windows, we find that models within 10-year time windows generally have higher prediction accuracy and higher consistency in predictions, and all the models also have good performances in predictions within 10-year time windows; from the perspective of models, we find that LSTM-HMM structures generally have good prediction

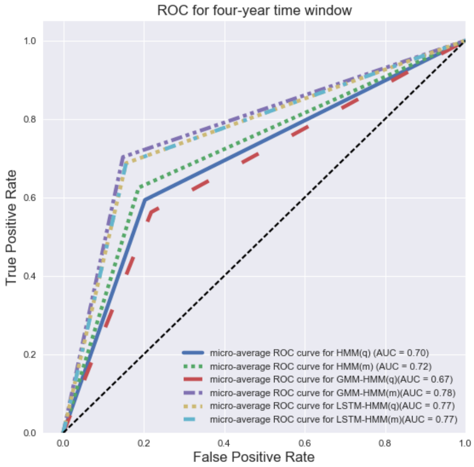

(a) 4-year time window

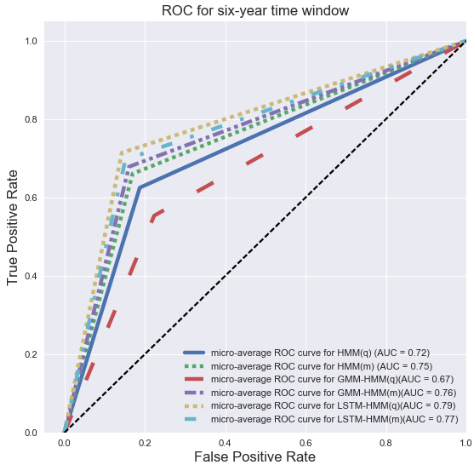

(b) 6-year time window

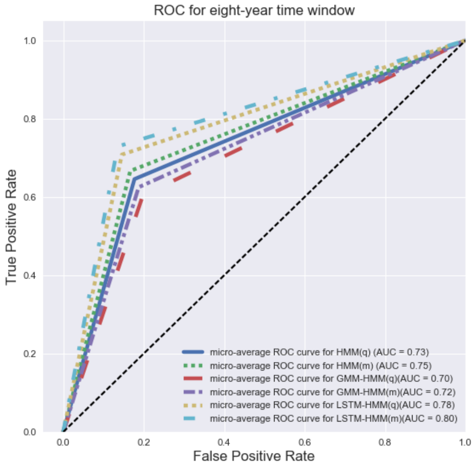

(c) 8-year time window

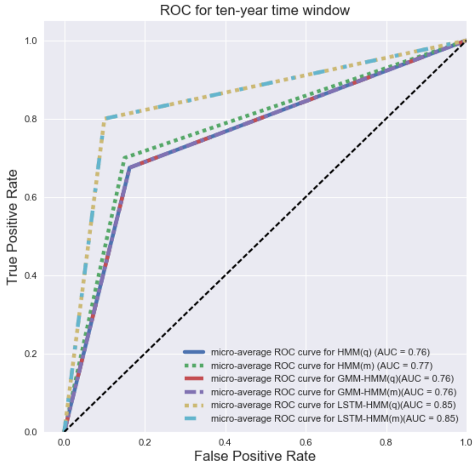

(d) 10-year time window

Figure 6: ROC for HMM, GMM-HMM and LSTM-HMM with monthly or quarterly input within 4-year, 6-year, 8-year and 10-year time windows

accuracy; from the perspective of overall forecasting effect, LSTM-HMM with either quarterly or monthly input within 10-year time windows has the best performance with the highest prediction accuracy and consistency; from the perspective of model input, when the model input transforms from quarterly to monthly, the model performance of the HMM, GMM-HMM and LSTM-HMM can generally be improved in terms of accuracy, kappa and AUC. .

There are several reasons for these model results: firstly, 10-year window with 40 observations in training is long enough for us to observe all types of GDP fluctuation states while avoiding the bias caused by too many observations of certain type as a proper data source for LSTM-HMM system; secondly, LSTM-HMM take more effects of historical CPI into the training process with the application of LSTM and tend to predict a suitable real time CPI fluctuation state, which replaces the lagged observable states adopted by other models, and helps in the prediction of GDP fluctuation using HMM; thirdly, since monthly data in the same season is more informative than quarterly data observed in the past season, the models with monthly input performs better than the models with quarterly input in most conditions.

Revised part on pages 19 and 20:

Firstly, we find that with an input of quarterly CPI, LSTM-HMM performs better within each time window. Secondly, we find that with an input of monthly CPI, GMM-HMM performs better within 4-year time window and LSTM-HMM performs better within 8-year time window, 8-year time window 10-year time window. Thirdly, among all the time windows, models within 8-year time window and 10-year time window have better overall performance in accuracy and consistency, which could avoid certain bias caused by too many observations of single type. HMM employed in our analyses depends on parameters calculated from sample data within certain time period, which means that these parameters change with time window and build different models. In the empirical analysis of GDP fluctuation states forecast, models encapsulated with HMM tend to ignore the effect of the sparse states and the giant difference of numbers of states will result in selection bias. In this paper, we reduce the impact of selection bias by experiments within pre-selected lengths of time window ranging from 4-year, 6-year, 8-year to 10-year and find that within 10-year window, the model performance is better. Fourthly, LSTM-HMM generally has the best accuracy and consistency, since this model predicts the real time CPI fluctuation states and take it as the observable state using LSTM, making the most of information conveyed by the monthly input and detecting useful historical information within a suitable time window.

Reviewer ♯1 OTHER COMMENT:
The paper is quite long with many small results that make it difficult for the reader to understand which are the most important to support the author's main claims. In order to improve in this aspect, I suggest that the main results should be highlighted for better readability.

Reply: Thank you very much for the suggestion. We highlight the main results to improve the readability. The revised parts are shown as follows:

Revised part on page 1:

This paper presents a Long Short Term Memory Recurrent Neural Network and Hidden Markov Model (LSTM-HMM) to predict China's Gross Domestic Product (GDP) fluctuation state within a rolling time window. We compare the predictive power of LSTM-HMM with other dynamic forecast systems within different time windows, which involves the Hidden Markov Model (HMM), Gaussian Mixture Model-Hidden Markov Model (GMM-HMM) and LSTM-HMM with an input of monthly Consumer Price Index (CPI) or quarterly CPI within 4-year, 6-year, 8-year and 10-year time window. These forecasting models employed in our empirical analysis share the basic HMM structure but differ in the generation of observable CPI fluctuation states. Our forecasting results suggest that (1) among all the models, LSTM-HMM generally performs better than the other models; (2) the model performance can be improved when model input transforms from quarterly to monthly; (3) among all the time windows, models within 10-year time window have better overall performance; (4) within 10-year time window, the LSTM-HMM, with either quarterly or monthly input, has the best accuracy and consistency.

Revised part on page 2:

Motivated by the usefulness of HMM and LSTM in economic forecast and the sparsity of literatures on the application of LSTM-HMM in GDP forecast, this paper establishes a LSTM-HMM to predict GDP fluctuation states. We further compare the predictive power of LSTM-HMM with HMM and GMM-HMM using monthly CPI or quarterly CPI within different time windows. There are three innovation points. First, in LSTM-HMM, we innovatively utilise LSTM to predict real-time CPI fluctuation states while feeding this prediction into the forecast of real-time GDP fluctuation states. Second, we select the inflation indicator CPI as the model input and utilise available monthly and quarterly CPI respectively in the LSTM-HMM. The

GDP fluctuation states are determined based on the turning points of GDP growth rate estimated by a double-threshold auto-regression model, while the CPI fluctuation states are identified through the instrumental variable estimation of s threshold model based on GDP growth rate and CPI growth rate. Third, we find from the empirical analysis of China's GDP fluctuation states that among all the time windows of rolling prediction, models within 10-year time window have better overall performance in accuracy and consistence and LSTM-HMM generally has good precision. The overall performance can generally be improved as the model input transforms from quarterly to monthly.

References

Peter CB Phillips and Sam Ouliaris. Asymptotic Properties of Residual Based Tests for Cointegration. *Econometrica*, 69(58):165–193, 1990.

Jinquan Liu. Recognition of phases of china's business cycle and forecasting for the growth trend. *China Industrial Economics*, 01:32–39, 2008.

Mehmet Caner and Bruce E Hansen. Threshold autoregression with a unit root. *Wiley Online Library*, 69(6):1555–1596, 2001.
